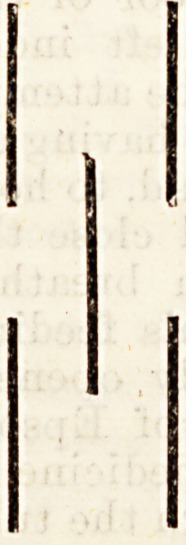# Treatment of Erysipelas

**Published:** 1893-12-09

**Authors:** 


### LONDON HOSPITAL.

### Treatment of Erysipelas.

Under the term erysipelas is included either several distinct diseases or several varieties of the same disease, classified usually into cutaneous, cellulocutaneous, and cellular erysipelas (or cellulitis). Whether having a different etiology, each variety arising from the presence of a separate micro organism, or not, the point of special practical interest lies in the fact that the clinical course, prognosis and treatment varies according to whether only the skin is involved, or the subcutaneous tissues in addition. When the skin only is implicated suppuration, with formation of abscesses, or tendency to necrosis, and sloughing of the subcutaneous tissue is very rare; on the other hand, when the connective tissue is affected, there is great danger of either the formation of localised abscesses or local death, sometimes of considerable portions of the subcutaneous tissues, forming sloughs which may take weeks in separating with loss of the skin over them, laying bare fascia and muscles, the resulting wound healing slowly by granulation. For the same extent of local affection the constitutional symptoms are more severe and the drain on the system greater than in the simple cutaneous variety.

Taking first the commonest form, simple cutaneous erysipelas, tending to recur after a first attack and probably in all cases starting from some wound or abrasion of the skin—the locality and extent are the first points examined, and the chief point of importance about the locality is when it affects the head and neck extensively, as it is then of more serious import than in any other part of the body. The greater the extent of surface involved, the more severe the attack, it being kept in mind that it is quite impossible to say in any given case how far the local manifestation of the disease will spread.

The other points upon which it is necessary to obtain information are the previous habits of the patient, especially with regard to alcohol, and the condition of the kidneys; chronic drunkards, or those in the habit of taking an excessive amount of alcohol, though they may never be actually drunk, are very liable to succumb to an attack of only moderate severity; and the same holds good for those whose kidneys are diseased, especially when the cirrhotic form of kidney disease, so often associated with abuse of alcohol, is present.

The treatment may be divided into the constitutional and local; the former, in anything like a severe case, being infinitely the most important. The product of the action of the micro-organisms of erysipelas on the tissues is a poison which causes death of the individual. When the dose produced and absorbed is greater than the body, as a whole, can recover from, and when the excretory system, especially the kidneys, are already inefficient, a smaller dose of the poison brings about a fatal result than when the organs are in an undamaged condition.

In the beginning of an attack the intestinal tract is cleared, usually by a dose of calomel or salts and senna. This has a double use—the absorption of food is promoted, and the tract placed in a good condition for the excretion of noxious substances from the blood. That a certain amount of the poison in the system is excreted by the intestinal tract is shown by the fact that diarrhœa is a not uncommon complication, and in some cases a severe and even fatal one. No drug has yet been discovered that will in any way cut short the course of the disease. Iron as the perchloride in full doses has often been credited with having this effect, but our experience with this drug does not show it to have any effect in altering either the course or severity of an attack. The drugs of most use internally, are stimulants in some form, to sustain the flagging action of the various important organs of the body, the heart especially being the one most apt to fail. Aromatic spirit or carbonate of ammonia, ether, alcohol, strychnine and digitalis are given, together with quinine or bark. The amount of alcohol, which is the best form of stimulant, being regulated by the rising of the pulse rate, and increasing feebleness of heart sounds, with restlessness and delirium. Opium or morphia in some form is given, unless there is much kidney disease, to soothe the pain and discomfort of the local lesion, or to lessen the delirium and restlessness. The point in which for many years past the treatment here has differed from that generally advised, is in the extensive use of cold as a local application. For this purpose the lotio plumbi is always used, a piece of lint covering the affected part and spreading edge is kept exposed to the air, and constantly wet with the cold lotion. This forms a very soothing application, easing the pain rapidly and efficiently; the only counter indication to its use is when a large extent of surface is involved in a very young child, as they do not bear well the extensive application of cold. The local applications advocated at one time or another are legion; those found most useful are the above, heat in some form or other, either as cotton wool simply bandaged on the part, or, better, moist heat as boracic fomentations or fomentations of hot saturated solution of hyposulphite of soda, this salt being believed by many to have a definite effect in shortening the course of the disease. Neither the application of solid nitrate of silver, or a solution of the same salt in water or nitrous ether, nor the hypodermic injection of carbolic acid, have been found to be of the slightest use in checking the spread of the local inflammation, nor has ichthyol used as an ointment been found of any use. Menthol cone rubbed in over the advancing edge and affected area of skin has often a definitely soothing effect in children,; sleep often follows its use, though not in any way influencing the course of the disease. Very rarely an abscess occurs in the affected area; nearly always after the local inflammation has begun to subside, and more often in children than adults; it is opened promptly and boracic fomentations applied. If opened freely a drainage tube is rarely necessary, the abscess being small and superficial.

The more severe forms of erysipelas, where the inflammatory process extends to the subcutaneous connective tissue, are associated with similar but severer constitutional symptoms, to which the same stimulant plan of treatment is applied, but in this form stimulants, preferably alcohol, have to be given with a more liberal hand, and for a longer time; food is given at frequent intervals, and usually in a fluid form, solids being rarely well taken. Cold as a local application is not so suitable as in the simple cutaneous form, moist heat as boracic fomentations being applied and the part affected kept at rest and slightly elevated. Watch is kept for the earliest signs of formation of pus, and even if there is no definite evidence of pus the tension of the inflamed part is considerably relieved by means of free incisions. These are made as a rule about two to three inches in length, and reaching well through the skin into the œdematous subcutaneous tissue, being arranged when possible parallel, and the ends of each incision over-lapping, giving complete relief to tension without making very extensive incisions. The flow of serum from the inflamed parts is encouraged by the frequent appplication of hot boracic fomentations, with the result that the tension is much lowered, injurious pressure is taken off the tissues and suppuration and necrosis prevented. The fomentations are continued, as long as there is any discharge, and as a rule the incisions are found to heal well under their use without any further treatment. Should pus have formed, or the tension have become so great before relief was afforded by incision that death of the subcutaneous tissue has taken place, free openings are made into the affected parts; if much suppuration is present, drainage tubes are passed from one incision to another, so that there shall be no collection of pus in the tissues. Boracic fomentations are applied, or, if the amount of suppuration and slough is very extensive, the part is kept in an antiseptic bath, either for four to eight hours a day, or else continuously. The baths used are boracic or weak perchloride of mercury, from 1 in 10,000 to 1 in 50,000, the object being to keep the part constantly clean rather than to act on it by any strong antiseptic. The treatment by bath is most useful when sloughing of the skin over the affected area is taking place, when it is used continuously at first, then alternated with hot fomentations to hasten separation of sloughs. When all the sloughs have separated, and there is little formation of pus, with healthy granulations over the whole or greater extent of the wound, the treatment becomes that of a granulating surface, and wet dressings of nitrate of silver or sulphate of zinc are used. Amongst the complications of erysipelas, one of the most important is severe diarrhœa, sometimes associated with vomiting and profuse sweatings, evidently due to absorption of a large dose of septic matter. When the diarrhœa is excessive opium with or without sulphuric acid and catechu is given, a moderate amount of diarrhœa not being checked. But in this condition alcohol or other stimulants are given freely with the opium, or the latter can be given as enema with a little starch, in which form it is most efficacious. During convalescence fresh air and good feeding are all important, with tonics, as quinine, iron and nux vomica, together with wine, beer, or stout at meal times, especially if the patient is previously accustomed to their use.

## Figures and Tables

**Figure f1:**